# Multimodal home-based rehabilitation intervention after discharge from inpatient geriatric rehabilitation (GeRas): study protocol for a multicenter randomized controlled trial

**DOI:** 10.1186/s12877-023-04634-2

**Published:** 2024-01-17

**Authors:** Bastian Abel, Martin Bongartz, Kilian Rapp, Patrick Roigk, Janine Peiter, Brigitte Metz, Benjamin Finger, Gisela Büchele, Michel Wensing, Catharina Roth, Oliver Schmidberger, Hans-Helmut König, Sophie Gottschalk, Judith Dams, Oliver Deuster, Désirée Immel, William Micol, Jürgen M. Bauer, Petra Benzinger

**Affiliations:** 1 Department of Clinical Gerontology, Robert-Bosch-Hospital, Stuttgart, Germany; 2 Center for Geriatric Medicine, Heidelberg University Hospital, Agaplesion Bethanien Hospital Heidelberg, Heidelberg, Germany; 3 Geriatric Center Karlsruhe, ViDia Christian Clinics Karlsruhe, Karlsruhe, Germany; 4 Department of Telemedicine, Robert-Bosch-Hospital, Stuttgart, Germany; 5 Institute of Epidemiology and Medical Biometry, Ulm University, Ulm, Germany; 6 Department of General Practice and Health Services Research, Heidelberg University Hospital, Heidelberg, Germany; 7 Department of Health Economics and Health Services Research, University Medical Center Hamburg-Eppendorf, Hamburg, Germany; 8 Interdisciplinary Center for Clinical Trials (IZKS) at the University Medical Center of the Johannes Gutenberg-University Mainz, Mainz, Germany; 9 AOK Baden-Württemberg, Statutory Health Insurance Company, Stuttgart, Germany

**Keywords:** Geriatric patients, Older adults, Physical capacity, Participation, Telemedicine, Tablet, Physical exercise, Physical training, Transitional care, Socio-medical, Post-ward, Nutrition, Multi-professional

## Abstract

**Background:**

Geriatric rehabilitation aims to maintain the functional reserves of older adults in order to optimize social participation and prevent disability. After discharge from inpatient geriatric rehabilitation, patients are at high risk for decreased physical capacity, increased vulnerability, and limitations in mobility. As a result, ageing in place becomes uncertain for a plethora of patients after discharge from geriatric rehabilitation and effective strategies to prevent physical decline are required. Collaboration between different health-care providers is essential to improve continuity of care after discharge from inpatient geriatric rehabilitation. The aim of this study is to evaluate the effectiveness of a multi-professional home-based intervention program (GeRas) to improve functional capacity and social participation in older persons after discharge from inpatient geriatric rehabilitation.

**Methods:**

The study is a multicenter, three-arm, randomized controlled trial with a three-month intervention period. Two hundred and seventy community-dwelling older people receiving inpatient geriatric rehabilitation will be randomized with a 1:1:1 ratio to one of the parallel intervention groups (conventional IG or tablet IG) or the control group (CG). The participants of both IGs will receive a home-based physical exercise program supervised by physical therapists, a nutritional recommendation by a physician, and social counseling by social workers of the health insurance company. The collaboration between the health-care providers and management of participants will be realized within a cloud environment based on a telemedicine platform and supported by multi-professional case conferences. The CG will receive usual care, two short handouts on general health-related topics, and facultative lifestyle counseling with general recommendations for a healthy diet and active ageing. The primary outcomes will be the physical capacity measured by the Short Physical Performance Battery and social participation assessed by the modified Reintegration to Normal Living Index, three months after discharge.

**Discussion:**

The GeRas program is designed to improve the collaboration between health-care providers in the transition from inpatient geriatric rehabilitation to outpatient settings. Compared to usual care, it is expected to improve physical capacity and participation in geriatric patients after discharge from inpatient geriatric rehabilitation.

**Trial registration:**

German Clinical Trials Register (DRKS00029559). Registered on October 05, 2022.

**Supplementary Information:**

The online version contains supplementary material available at 10.1186/s12877-023-04634-2.

## Background

Limitations in mobility and the activities of daily living (ADL) are highly prevalent in older adults and may lead to a loss of independence in this population [1, 2]. A decrease in muscle mass, muscle strength, and flexibility as well as impairments in balance and coordination are likely to compromise the functionality and mobility of older people. Acute illness and hospitalization pose a particular threat to the mobility of older adults and may precipitate a decline in functionality and an increase in the risk of admission to long-term care facilities [3]. Hence, strategies to maintain mobility and independence are of great relevance to foster quality of life in older adults.

Rehabilitation is a key strategy to achieve the third of seventeen Sustainable Development Goals of the World Health Organization (WHO): "ensure healthy lives and promote well-being for all at all ages" [4]. The WHO defines rehabilitation as “a set of interventions designed to optimize functioning and reduce disability in individuals with health conditions in interaction with their environment” [5]. Rehabilitation addresses limitations in daily physical, mental and social functioning caused by aging or an acute or chronic illness. Geriatric rehabilitation (GR) aims to promote activity and maintain the functional reserves of older adults to optimize social participation and to prevent disability [6]. While there is a growing demand for GR [4], access to and duration of GR services are limited due to financial restraints in most health-care systems [7].

In its implementation, GR follows a multidimensional approach including diagnostic and therapeutic measures. Physical exercise is a cornerstone of GR and focuses on improving mobility, endurance, balance, and strength, which are relevant predictors for independent living [8–11]. Even though several months of exercise are necessary to most effectively improve balance and strength [12, 13], the average length of stay in inpatient GR is limited to three weeks throughout Europe [6]. In Germany, GR is predominantly provided as an inpatient post-acute program and the planned length of stay in the rehabilitation department is about three weeks [14]. Given their limited timeframe, most GR programs are insufficient to achieve exercise-induced long-lasting physical adaptations. A sustained physical response to exercise can only be achieved with regular, long-term physical exercise [14, 15]. For a continuous improvement in functional capacity, patients should be empowered to exercise regularly after discharge from inpatient GR [16]. The effectiveness of continued exercise at home after discharge from inpatient rehabilitation has been well established [17–22]. To restore social participation, the complex needs and limitations of patients discharged from GR require a multi-professional model of care. This model of care must go beyond the provision of exercise to address the immediate needs of newly discharged patients through close collaboration between all health-care professionals, caregivers, and patients.

According to the International Classification of Functioning (ICF), overcoming social and environmental barriers is equally relevant to regain the previous levels of mobility and participation [23]. To fulfill its commitment to counteract participation restrictions, GR needs to consider the patients’ contextual aspects when returning to their living environment. Discharge to the community should warrant tight collaboration with families, community services, and primary care physicians to ensure adequate social support and medical care. However, discharge planning during inpatient care is often based on several assumptions regarding the living and care situation, and subsequent adjustments are often necessary. For example, the removal of tripping hazards and barriers to outdoor mobility can rarely be initiated by hospital-based health-care professionals during inpatient care.

Besides contextual factors, nutrition is of high relevance for the functional improvement of patients in GR. Since malnutrition and the risk of malnutrition are highly prevalent in GR patients [24], most GR facilities provide nutritional support [25, 26]. After discharge to the community, financing of a protein and energy supplementation might not be covered by the health-care system and pragmatic interventions may be necessary.

In Germany, medical and nursing care is performed by public and private providers, and citizens are largely free to choose their provider. The delivery and reimbursement of inpatient and outpatient medical care is strictly separated, which causes problems particularly in bridging the gap between hospital and outpatient care. Due to the separation of inpatient and outpatient care, the responsibility of hospitals ends upon discharge and adjustments after discharge are at the discretion of the patients. Therefore, health insurance companies are mandated to support discharge planning [27, 28]. In practice, hospitals and health insurance companies mutually complain about organizational problems in their collaboration [27, 29]. Especially for complex cases, models of structured collaboration between hospitals and health insurances are needed and digital solutions inhere the chance to overcome communication barriers.

The aim of this three-center study is to establish a three-month multimodal home-based intervention program following discharge from inpatient GR. The GeRas program *(“Geriatric Rehabilitation success”) *is a multi-professional intervention that includes individually adjusted progressive physical exercises, social counseling, and nutritional recommendation, initiated during the inpatient treatment and continued through a structured handover in the outpatient setting. The intervention will be delivered either conventionally by home visits and telephone calls (conventional intervention group, conventional IG) or based on an eHealth system by using tablet computers and a combination of home visits and video calls (tablet IG). The primary study aim is to evaluate the effectiveness of the GeRas program on the lower extremity function and, additionally, the social participation after three months. Secondary study aims will focus on the primary outcomes after six months, physical activity, frailty, concerns about falling, falls, movement-related self-efficacy, life-space mobility, nutritional status, health status, and health-related resource use. Furthermore, the costs and cost-effectiveness of the GeRas program will be analyzed, the tablet IG and the conventional IG compared, and barriers and facilitators for the implementation into routine care identified.

## Methods

### Study design

This study is a three-center, assessor-blinded, randomized (1:1:1), controlled, parallel-group trial with a three-month intervention period and three-month follow-up period. Two hundred seventy participants will be recruited from inpatient GR at three study sites: (i) Robert-Bosch-Hospital Stuttgart, Germany (ii) AGAPLESION BETHANIEN HOSPITAL HEIDELBERG, Germany, and (iii) ViDia Christian Clinics Karlsruhe, Germany. This study protocol was prepared in accordance with the SPIRIT (Standard Protocol Items: Recommendations for Interventional Trials) guidelines [30].

### Eligibility criteria

Eligible participants are patients receiving inpatient GR, discharged to the community or assisted living, able to walk independently with or without a walking aid at room level, and insured with the Allgemeine Ortskrankenkasse (AOK) Baden-Württemberg (largest statutory health insurance company in the German federal state of Baden-Württemberg). All inclusion and exclusion criteria are listed in Table 1.

**Table 1:** Inclusion and exclusion criteria for study participation

Inclusion criteria	Exclusion criteria
• inpatient geriatric rehabilitation • health insurance with AOK Baden-Württemberg • walking ability indoors with or without use of a walking aid • discharged home or to assisted living • residence within the catchment area of the study site (≤ 30 km or 1 h travel time by public transport)	• planned inpatient treatment within the following 3 months after discharge • outpatient/mobile rehabilitation following inpatient rehabilitation • institutional care (permanent/respite/short-term care) after discharge • instable medical conditions that do not allow physical training • terminal phase of a disease (life expectancy < 6 months) • moderate to severe cognitive impairment (MMSE < 21) • insufficient German language skills • hearing not sufficient to communicate via telephone • visual acuity not sufficient to recognize study material

*AOK* Allgemeine Ortskrankenkasse (German statutory health insurance company), *MMSE* Mini-Mental State Examination

### Components of the intervention program

The participants in both IGs will receive a three-month multimodal home-based intervention program that aims to improve mobility and to improve social participation. The GeRas program begins upon discharge from inpatient GR, ends after 13 weeks, and will be delivered by a multidisciplinary team consisting of physical therapists, geriatricians, and social workers employed at the discharging GR as well as social workers employed at the AOK Baden-Württemberg. Key components of the GeRas program are a) a physical exercise program, b) social counseling, c) person-environment fit, and d) nutrition advice. The exercise program and the nutrition counseling will be delivered by health-care professionals of the discharging GR, namely physical therapists and geriatricians, respectively. The social workers of the GR forward the participants’ social and care needs in detail to the social workers of the health insurance, who will render outpatient social counseling. Delivery of the program will be monitored by geriatricians, but the medical treatment will be left at the discretion of the participants’ primary care physician, who receives a detailed discharge report and records of the participants’ progress.

Participants’ mobility-dependent goals will be agreed at discharge from inpatient GR and both IGs will receive the same program content. Only the mode of delivery will differ between the two IGs.

**Fig. 1:**
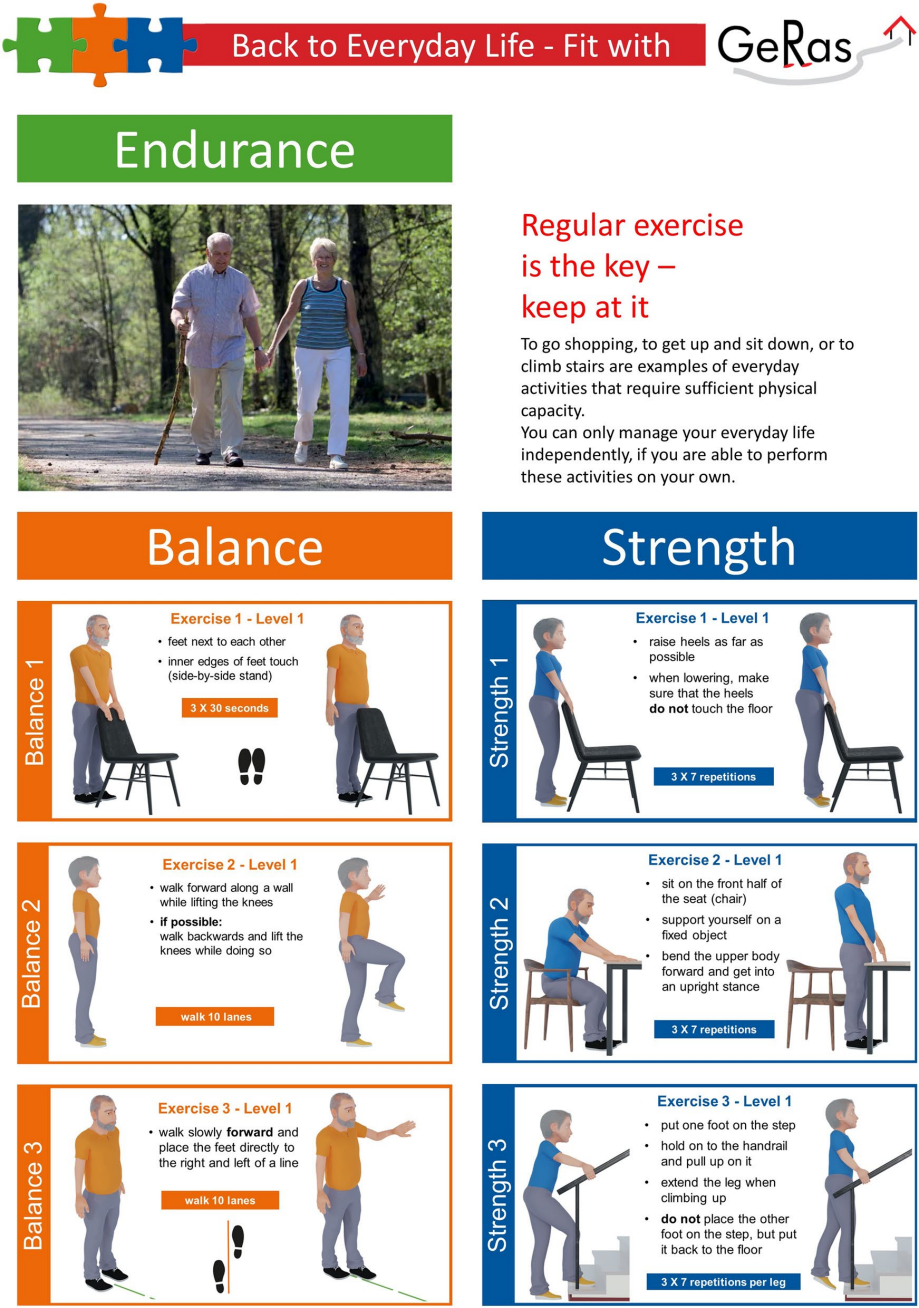
Exercise poster of the conventional intervention group (exemplified with the easiest level of difficulty)

*Conventional IG*: a) the exercise intervention will be delivered through five home visits by the physical therapists (scheduled in weeks 1, 2, 4, 7, and 11 after discharge), a poster (Fig. 1) and a manual illustrating the exercises to support an autonomous training by the participants, and weekly telephone calls by the physical therapists to promote motivation and adherence to exercise. b) The social workers of the health insurance will contact the participants by telephone in the first few days after discharge, inquire about immediate unmet care needs, and schedule a follow-up home visit. If needed, socio-legal steps will be initiated to improve care. c) All needs for assistive devices and adjustments of the home environment (removal of home hazards and possible home modifications) will be assessed by the social workers of the health insurance and the physical therapists. In addition, social workers of the health insurance will be responsible for counseling on home modifications. d) The nutritional status of participants will be assessed after six weeks based on weight change, appetite, and adequacy of protein intake. Participants will receive written information on their status alongside recommendations to optimize their nutrition.

**Fig. 2:**
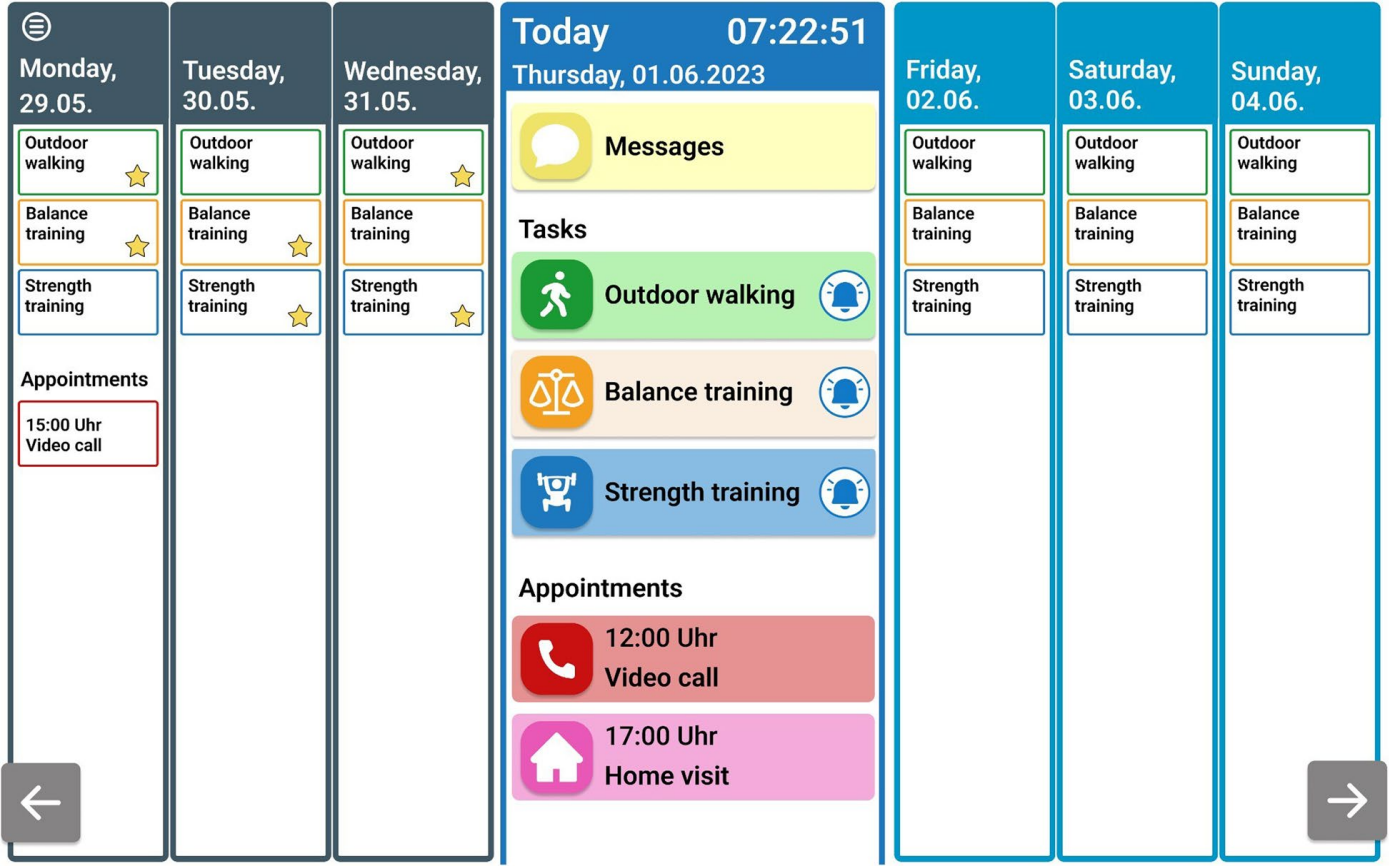
Calendar view on the tablet

*Tablet IG*: The intervention will be delivered through home visits and video calls using a tablet computer provided by the research group, simplified in its use, and adapted for older people. a) The participants will receive two home visits (at weeks 1 and 2 post-discharge), three video consultations (at weeks 4, 7, and 11 post-discharge), as well as weekly motivational video calls by physical therapists. The telemedicine application used on the tablet computers to conduct the intervention is part of the “Curafida” telemedicine platform [31] and was developed specifically for and in collaboration with representatives of the target group. The application is designed as a kiosk software and mostly limited to the features that are necessary for the tablet IG. With as few clicks as possible, the participants can view their personal training schedule, watch videos of the selected exercises, and view appointments with their physical therapists and social workers in a calendar view (Fig. 2). These features replace the poster and manual used in the conventional IG. An application enabling video calls is linked to the calendar view and participants do not need to start any further applications themselves. During video consultations, participants demonstrate the execution of exercises and receive feedback on their progress from the physical therapists. To facilitate the demonstration of exercises by the participants, the tablets will be delivered alongside an adjustable tablet stand. At the same time, physical therapists can adjust the exercise program displayed and participants’ training schedule remotely. b) Scheduled home visits by the social workers will be replaced by video consultations, unless the social workers deem home-visits necessary. The nutritional advice and the person-environment fit adaptations will be delivered equivalently to the conventional group.

The interdisciplinary exchange of information and management of the participants is realized within a cloud environment as part of the “Curafida” telemedicine platform and specifically adapted to the needs of GeRas.

### Content of the intervention

The following is a brief description of the individual components of the intervention.

- *a) Physical exercise program*

The home exercises are based on a structured program that has been evaluated previously [20]. No later than 10 days after discharge from inpatient GR, the first home visit will take place, during which the exercise program will be individually adapted and practiced. The program consists of three exercises to strengthen the lower limbs and three exercises to train the participants’ balance. Each exercise is available in three levels of difficulty to enable a progression of the training (see supplementary material). The appropriate level of difficulty is based on the participants’ goals, adapted to their individual physical abilities, and determined by the therapists. In addition, a walking course within the participants’ neighborhood is agreed upon to improve the participants’ physical endurance. The second home visit will take place the following week to review and correct any mistakes. The participants are encouraged to complete all exercises once per day. Supervision of the training program by the physical therapists is performed through a total of five home visits and weekly telephone calls (conventional IG) or two home visits, three video consultations, and weekly video calls (tablet IG). To encourage and evaluate adherence to exercise, the participants are asked to keep a training diary.

The participants of the tablet IG can see their exercises as daily tasks in their calendar view (Fig. 2). Once clicked and marked as successfully completed, the participants automatically receive motivational feedback and a small bell icon in the calendar view changes to a star icon. The tasks marked as completed allow the physical therapists to remotely monitor exercise compliance.

The difficulty of the exercises will be gradually and individually adjusted by the physical therapists as the physical capacity changes over the course of the intervention. This progress can be monitored and controlled based on the exercise completions recorded by the participants. In addition, weekly telephone or video calls are used to increase motivation and to record falls or other adverse health events.

- *b) Social counseling*

The social workers of the GR will provide the social workers of the health insurance with information about functional limitations, the social and living environment, identified needs, as well as their interventions. They will also report on whether any next of kin were involved in the counseling at the hospital and, if so, who they were.

The social workers of the health insurance will provide post-discharge support on issues related to community care and outpatient medical care, financial support for medical and care services as well as assistive devices, and will monitor the execution of the measures subsequently. They will identify unmet needs and initiate the involvement of care services as needed. Their first counseling will be via telephone two to six days after discharge in order to identify unmet urgent needs - i.e. whether the interventions initiated by the GR are being received or whether other needs require further intervention (e.g., provision of assistive devices, application for financial support, involvement of community care services, provision of prescriptions). Depending on further needs and group allocation, the participants will either be visited at home once or twice during the intervention period and called two to three times (conventional IG), or contacted three to five times via video consultation (tablet IG). If necessary, next of kin are involved in the counseling process. Two to three weeks after discharge, the social workers of the health insurance will conduct a home visit (conventional IG) or video consultation (tablet IG) to check whether the measures initiated thus far are being successfully implemented and whether all needs are being covered. As part of the counseling, barriers to mobility will be analyzed and, if necessary, measures to improve social participation will be initiated (e.g., prescription of assistive devices, recommendations on home adaptations, initiation of social support services). After seven weeks, the social workers of the health insurance will contact the participants again by telephone or video call and will inform them about local participation activities providing social contact and/or physical activity (e.g., suitable sports activities, community service clubs). During the counseling process, barriers to participation in these activities, such as financing or transportation issues, will be discussed.

- *c) Person-environment fit*

Identifying the need for a person-environment fit will be a two-step process. First, during the initial telephone consultation, the social workers of the health insurance will use a self-developed checklist to inquire about barriers in the home environment and in the outdoor space, as well as the need for assistive care, mobility devices, and home modifications. Second, the physical therapists will look for tripping hazards in the home and barriers to safe outdoor mobility during their first home visit; they may make recommendations to the participants and involve social workers of the health insurance in order to provide participants with assistive devices or to counsel on financial support for home modifications.

- *d) Nutrition advice*

Participants will be asked about their weight and fluid status by physical therapists at five to six weeks after discharge. In addition, appetite and protein intake will be assessed using the SNAQ and Protein Screener (Pro55 +), respectively [32, 33]. The Protein Screener is a validated online tool to assess adequacy of protein intake based on the recommendation of a protein intake ≥ 1.0 g/kg of body weight per day. Geriatricians consider this information, along with known diagnosis and weight change during and after rehabilitation, to provide an individualized written recommendation on healthy food alongside a brochure of suggested high-energy, high-protein foods.

### Coordination of the program

The geriatricians of the discharging GR coordinate all professionals involved in the delivery of the intervention. They coordinate online multidisciplinary post-discharge case conferences with physical therapists and social workers (two to three weeks and six to seven weeks after discharge) and provide brief reports of identified medical concerns to the participants' primary care physicians. If necessary and only with consent, they contact the primary care physicians by telephone.

### Control group

The participants assigned to the CG will receive usual care from their primary care physicians as well as routinely available health and older adult care services from the German health-care system. This may include outpatient physical therapy, if deemed necessary by the primary care physicians, and counseling by the AOK social services, if requested by the AOK Baden-Württemberg members. To decrease attrition, two short handouts on general health-related topics are sent to the participants in week 4 (covering diet, sleep, and relaxation) and week 9 (physical activity and fall risk) after discharge. The participants will receive a brochure from the German Nutrition Society (Deutsche Gesellschaft für Ernährung e.V., DGE) with general recommendations for a healthy diet (10 guidelines of the DGE for a wholesome diet [34]) and a lifestyle counseling with general advice for active ageing, delivered by physical therapists (at the participants’ home after completion of the T1 assessment).

### Outcomes

The primary and secondary outcomes, as well as descriptive variables, are shown in Table 2. All primary outcomes will be assessed at participants' homes, whereas some secondary outcomes and descriptive variables are recorded at the end of inpatient rehabilitation.

**Table 2:** Overview of the primary and secondary outcome measures, descriptive variables, and date of data collection

	**Variables**	**T**_**0a**_	**T**_**0b**_	**IA**^**a**^	**T**_**1**_	**IA**^**b**^	**T**_**2**_
**Sociodemographics**							
D	Age, sex, education, living conditions	x					
**Medical information & comorbidity**							
D	Diagnosis relevant for rehabilitation	x					
D	Cumulative Illness Rating Scale-Geriatric [35]	x					
D	Pain		x		x		x
D	Intercurrent medical events (diseases, accidents)		x	x^a^	x	x^b^	x
D	Medication (type, dosage, frequency)		x		x		x
**Health-related quality of life and resource use**							
S	EuroQol-5-Dimension 5-Level [36]		x		x		x
S	Adapted version of the questionnaire for the use of medical and non-medical services in old age (FIMA) [37]				x		x
**Information on general physical health condition, body weight, and nutritional & gastrointestinal status**							
S	Simplified Nutrition Appetite Questionnaire [38]	x	x	x	x		x
S	Mini Nutritional Assessment—short form [39]		x		x		x
D	Global Leadership Initiative on Malnutrition criteria [40]	x					
D	Subjective Global Assessment [41]	x					
**Anthropometric data**							
D	Body weight	x	x		x		x
D	Ulnar length		x				
D	Arm circumference		x		x		x
**Frailty**							
S	Groningen Frailty Indicator [42]		x		x		x
S	Clinical Frailty Scale [43]		x		x		x
**Neuropsychological status**							
D	Trail Making Test (A & B) [44]		x				
**Falls, concerns about falling, and management of falls**							
S	Falls Efficacy Scale International - short form [45]		x		x		x
S	Perceived Ability to Manage Falls [46]		x		x		x
S	Number of falls		x	x^a^	x	x^b^	x
**Social participation, mobility, physical capacity, and activity**							
P, S	Modified Reintegration to Normal Living Index [47]		x		x		x
S	Late-Life Function and Disability Instrument – short form (disability component) [48, 49]		x		x		x
S	Life-Space Assessment for Persons with Cognitive Impairment [50, 51]		x		x		x
P, S	Short Physical Performance Battery [52]		x		x		x
S	Handgrip strength [53]		x		x		x
S	Physical activity assessed with body-worn sensors		x		x		x
**Goal attainment and training adherence (only in intervention groups)**							
D	Goal Attainment Scale [54]				x		
D	Training diary				x		x
**Technology experiences**							
D	Experience with the use of smartphones, tablets, or laptops		x				
D	Mobile Device Proficiency Questionnaire-16 [55]		x				
D	System Usability Scale [56]				x^c^		

*Abbreviations*: *P* Primary outcome measure, S Secondary outcome measure, D Descriptive measure, T_0a_ Assessment by clinical physician, T_0b_ Baseline assessment, T_1_ 3-month assessment, T_2_ 6-month assessment, IA Interim assessment (^a^ within the intervention period, ^b^ within the follow-up period)

^a^recorded once a week, ^b^recorded once a month, ^c^only in the tablet intervention group

The assessments will be distinguished as follows: T0a = assessment by a physician (1–5 days before discharge), T0b = baseline assessment (2–6 working days after discharge), T1 = three-month post-assessment (13 ± 1 week[s] after discharge), and T2 = six-month follow-up-assessment (26 ± 2 weeks after discharge).

#### Primary outcomes

The primary outcome measures will be the physical capacity measured by the Short Physical Performance Battery (SPPB) and the social participation assessed by the modified Reintegration to Normal Living Index (mRNLI), 3 months after discharge (T1).

The SPPB is an objective assessment tool for evaluating lower extremity function in older persons including static balance, gait speed, and repeated chair rises [52]. It is considered a "gold standard" for evaluating exercise interventions in older persons, as the result is highly predictive for disability, rehospitalization, institutionalization, and mortality [57–60], and therefore a good indicator for future loss of autonomy.

The mRNLI is an instrument that incorporates social, psychological, and physical aspects and assesses patients’ perceptions of their ability to resume activities of daily living after a disabling illness or injury [47]. This 11-item questionnaire consists of two subscales. Information about regular activities, including questions about mobility, ability for self-care, daily activities, leisure activities, social activities, and family roles, is summarized in the subscale “Daily Functioning”. Interpersonal and intrapersonal behaviors, represented by questions on personal relationships, self-presentation, and general coping skills, are summarized in the subscale “Personal Integration”. All items of the mRNLI are rated on a 4-point Likert-scale (0 points = does not describe me or my situation; 1 point = sometimes describes me or my situation; 2 points = mostly describes me or my situation; 3 points = fully describes me or my situation). The mRNLI has demonstrated validity in studies of community integration of persons living with long-term consequences of chronic health conditions [61] and has been validated in pre-frail to frail older persons living in the community [62].

#### Secondary outcomes

Lower extremity function, assessed with the SPPB, and the self-perception of the ability to resume activities of daily living after the inpatient rehabilitation, measured with the mRNLI, will also be considered as secondary outcomes at the end of the follow-up period (T2).

Concerns about falling during social and physical activities will be assessed with the short form of the Falls Efficacy Scale International (short FES-I) [45]. Elements of self-efficacy independent of physical activities and the ability to cope with falls will be assessed with the Perceived Ability to Manage Falls scale (PAMF) [46].

Frailty will be assessed by a self-assessment instrument (Groningen Frailty Indicator; GFI) [42] as well as the Clinical Frailty Scale (CFS) rated by assessors [43].

Information on the frequency of participation in particular activities and the associated limitations on engaging in those activities will be measured with the disability component of the Late-Life Functional and Disability Instrument (LLFDI) [48, 49].

The Life-Space Assessment in Persons with Cognitive Impairment (LSA-CI) will be used to assess the participants’ life-space mobility. The LSA-CI is an instrument to assess life-space mobility of the previous week by the frequency of movements and assistance needed to reach the individual activity area [50, 51].

Physical activity will be objectively recorded over 7 days using a 6-axis inertial measurement unit (AX6, Axivity Ltd., Newcastle, UK) fixed at the lower back. Handgrip strength will be measured with a JAMAR digital hand dynamometer following a standardized protocol [53].

The participants' appetite will be determined by using the Short Nutritional Appetite Questionnaire (SNAQ). This four-item questionnaire is a validated and reliable instrument, which is easy to use in older adults [38]. The nutritional status will be assessed with the short form of the Mini Nutritional Assessment (MNA-sf), a validated assessment tool to detect malnutrition in geriatric patients [39]. On discharge from inpatient rehabilitation, malnutrition will be diagnosed according to the Global Leadership Initiative on Malnutrition (GLIM) criteria as well as by the Subjective Global Assessment [40, 41].

To quantify the participants’ health-related quality of life, the 5-level version of the EuroQol-5-Dimension questionnaire (EQ-5D-5L), including a visual analog scale to quantify subjective overall health, will be used [36].

Pain at rest and while walking or standing will be assessed using a 5-point verbal rating scale.

#### Descriptive variables

Morbidity will be assessed by the primary diagnosis on admission to rehabilitation as well as the Cumulative Illness Rating Scale in its adaptation to geriatric patients (CIRS-G). This scale covers 13 somatic domains as well as one psychiatric domain [35].

Furthermore, hospital readmissions including the reasons for rehospitalization and the duration of inpatient treatment as well as the number of falls and fall-related injuries will be recorded by weekly and monthly interviews via telephone during the intervention (week 1 to 12 ± 1) and follow-up period (week 13 to 26 ± 2), respectively.

The cognitive status of the participants will be evaluated by using the Trail Making Test A + B (TMT). The TMT provides information on visual search, scanning, speed of processing, mental flexibility, and executive functions [44].

Goal Attainment will be evaluated with the Goal Attainment Scale [54] after the twelve-week training intervention (T1).

The experience with the use of smartphones, tablets, or laptops will be documented. To evaluate the usability of the telemedicine application, the System Usability Scale, a 10-item questionnaire with a 5-point Likert scale, will be used in the tablet IG [56]. The Mobile Device Proficiency Questionnaire-16 (MDPQ-16) will be used to assess basic and advanced proficiencies related to tablet and smartphone use across different subscales. The participants will be asked to rate their ability to perform 16 operations on a tablet device or a smartphone on a 5-point Likert-scale [55].

### Health economic evaluation

A cost-effectiveness analysis will be conducted after six months (T2). Intervention costs as well as health care utilization costs will be considered for the evaluation. All costs directly related to the intervention will be estimated with personnel costs based on the average German wages of the professional groups involved and their time spent on the intervention. Participants’ utilization of health-care services will be assessed with an adapted version of the FIMA questionnaire (German: ‘Fragebogen zur Inanspruchnahme medizinischer und nicht-medizinischer Versorgungsleistungen im Alter’) [37]. This questionnaire considers both medical services (e.g. inpatient and outpatient treatment, medications, rehabilitation services) as well as formal and informal care (e.g. assistance with activities of daily living by relatives, neighbors, or friends). Service use will be collected retrospectively for three months at T1 and T2. Costs will be calculated by monetary valuation of service use using standard unit costs [63]. For the cost-effectiveness analysis, health effects will be measured by quality-adjusted life years (QALYs) [64] based on the EQ-5D-5L [65]. Incremental cost-effectiveness ratios (ICER) will be calculated as the ratio between the difference in mean costs and the difference in mean effects. To account for statistical uncertainty, cost-effectiveness acceptability curves will be constructed based on net benefit regressions [66].

### Process evaluation

In order to evaluate the implementation of the GeRas program intervention and the success of continuity of care, a process evaluation will be conducted using interviews and questionnaires in health-care providers, patients, and intervention developers. The process evaluation aims to assess the intervention in terms of effectiveness, scalability, and sustainability. This process evaluation is based on the Consolidated Framework for Implementation Research, a recognized framework in implementation research [67]. It will be examined, a) whether the intervention of the GeRas program is implemented as intended (intervention fidelity), b) whether the intervention needs to be adapted (adaptation), c) to what extent the intervention of the GeRas program is implemented (dose), d) whether the potential addressees are reached (reach), and e) how participants evaluate the intervention and whether they adhere to the program (participant response). Furthermore, the experiences and perceptions of health-care providers involved in the implementation of the intervention will also be evaluated. The process evaluation will help to identify significant barriers and facilitators for successful implementation (context factors) and later transferability of the intervention into standard care (sustainability and transferability). Data will be collected based on a mixed-methods approach using a paper-based survey and semi-structured interviews. The participants will be asked to complete a brief paper-based survey and to participate in an interview after the completion of the intervention period. Health-care providers will be interviewed throughout the implementation period in order to capture early and later experiences and will be asked to complete a paper-based survey at the beginning and the end of the implementation period.

### Participant timeline

All patients will be screened for eligibility during the first two weeks after admission to inpatient GR and approached for participation if eligible. Following their informed consent, patients will be randomly assigned to one of the two IGs or the CG three to five working days before discharge. The baseline assessment will be completed within two to six working days (Monday to Friday) after discharge. The patients assigned to the intervention groups will be contacted by social workers of the health insurance after completion of the baseline assessment and no later than six days after discharge. The intervention period ends 13 weeks after discharge from GR. In order to harmonize the recording of falls and intercurrent medical events between the groups, all participants will be contacted weekly during the 13 weeks after discharge. These contacts will be conducted either as part of the scheduled contacts with the physical therapists (IGs) or through telephone calls explicitly asking about falls and medical events (CG). During the follow-up, all participants will be contacted monthly by telephone. Further assessments will be performed 3 months and 6 months after discharge. All assessments will last about 90 min each.

### Sample size

A power estimation was performed for the comparison of conventional IG vs. CG with differences in SPPB total score between T0b and T1. Information on changes within the control group and standard deviations is taken from a previous study [21]. A minimal clinically significant effect was defined as 1 point of the SPPB total score according to Perera et al. [68]. Assuming a recruitment of 90 participants in each study center will result in 270 participants in total within the recruitment period. At the study end, 182 participants (distributed among the three study groups) would be left for analyses after deducting 10% of participants due to a potentially higher variance in a multicenter design and an additional 25% due to the expected number of dropouts. This number of participants is sufficient to achieve a power of > 80% at a significance level of 5% using a 2-tailed *t*-test regarding the mentioned effect.

### Recruitment

After admission to inpatient GR, potentially eligible participants will be identified by means of a pre-screening (health insurance with AOK Baden-Württemberg, residence within the catchment area of the study site). The patients' eligibility will be further ascertained by a geriatrician according to inclusion and exclusion criteria (Table 1). If the patients are eligible and willing to participate, the geriatrician or the local study coordinator will obtain written informed consent from the patients.

### Randomization and blinding

Participants will be randomly assigned to one of the IGs or the CG using computer-generated block randomization with a 1:1:1 allocation ratio stratified by study site. The randomization will be performed by the Institute for Epidemiology and Medical Biometry of Ulm University, Ulm, Germany, using the randomization program ROM [69]. All assessments of primary and secondary outcomes will be performed by assessors blinded to group allocation. Data that reveal group allocation (e.g., training diaries) will be collected by the unblinded physical therapists prior to T1.

### Data collection and management

A database management system (in-house developed based on TeleForm® and MS-Access®) with components both on-site at the study center and at the data management center will be used for data collection and management. For the baseline and follow-up assessments at the participants' homes, an electronic case report form with programmed plausibility checks will be used to be completed offline and later uploaded to the database. Paper-based case report forms or documents (e.g. medical discharge questionnaires, training diaries, or process evaluation questionnaires) will be used in a machine-readable format and the collected data will be integrated into the database after verification. The on-site database management system includes automatic functions for reminding data collection and detecting missing or implausible data entries. This approach minimizes errors and missing data and ensures a high quality of data collection. Individual and identifiable information of the participants will only be stored in the on-site database as well as on encrypted local servers at the respective study site. Intercurrent events (falls, adverse events) in all groups will be collected by the physical therapists, entered on the telemedicine platform, and forwarded to the database manager via the telemedicine provider. All other data entries and contents of the telemedicine platform can be integrated into the database via the same path, if required. Only authorized study personnel and the external clinical monitor will have access to the database. All pseudonymized data will be collected at the data management center and the final data set will be accessible only to study personnel directly involved in the data analysis.

### Statistical analyses

A pairwise comparison of the primary study endpoints (SPPB and mRNLI at T1) will be performed. The comparison to the SPPB between conventional IG and CG will be analyzed in a confirmatory manner and considering the intention-to-treat principle. Further potential comparisons of these outcomes between the groups or time points or considering covariates will be evaluated exploratory using, for example, multivariate regression models. The secondary endpoints will be analyzed exploratory as group comparisons at T1 and T2 or over time using the appropriate statistical methods (e.g. statistical tests or multiple regression analyses further specified in a statistical analysis plan before analysis).

### Data and safety monitoring

The study is monitored by external clinical monitors (Interdisciplinary Center Clinical Trials, Mainz University Medical Center, Germany), independent of the institutions and investigators involved in the conduct of the intervention. Systematic quality assurance and control according to a monitoring plan includes periodic on-site visits at each study site, risk-based review of source data, and site management. To control participant risk and assess study safety, (serious) adverse events (S[AE]) and adverse events related to falls (adverse events of special interest [AESI]) will be monitored by the study team throughout the study. An SAE is defined as any harmful disease or injury that requires inpatient hospitalization, prolongation of inpatient hospitalization, is life-threatening, or results in death or significant and permanent disability or incapacity [70]. Intercurrent medical events and falls of the participants will be continuously recorded every week during the three-month intervention period (T0b-T1) and every month during the subsequent follow-up period (T1-T2). This collects information about the date, time, and location of the falls, as well as activities prior to the fall and any injuries that occurred. The severity of falls will be categorized according to Schwenk et al. [71]. All SAE and AESI will be reported to the principal investigator. The principal investigator reviews and reports SAE to the sponsor. All study participants are covered by an insurance.

## Discussion

Regaining independence, mobility, and participation are important goals of GR and essential for aging in place. The majority of patients indicate mobility-related functions as the most important rehabilitation goals at admission [72, 73]. Returning home after discharge from rehabilitation is often considered as the primary indicator of a successful rehabilitation process from the patients' perspective [10, 72]. Although functional capacity can be further improved by exercise interventions after returning to the home environment [20], the previous levels of independence and social activity are often neglected. During the initial post-discharge period, patients are temporarily at general risk for a number of adverse health events that prevent structured exercise interventions [74]. Patients report a variety of problems after discharge, e.g. difficulties with self-care, and the need for help because they are unable to solve these problems [75]. Even a structured discharge planning, which may diminish or prevent some of these problems, does not improve the post-discharge functional status without ongoing exercise [76].

The GeRas study will evaluate a novel program that provides continuity and coordination of care in the transition phase after discharge from an inpatient GR facility to the home environment [77]. The program will provide rehabilitation support after discharge from inpatient GR to the community in a step-down strategy, a supportive and rehabilitative health care given to patients recuperating from an illness and regaining autonomy. Following the holistic approach of inpatient GR, the novel GeRas program will focus specifically on mobility and mobility-related participation restrictions. The program would be suitable for rapid implementation into existing health care structures. Physical therapists, social workers, and geriatricians will support the patients over a period of three months in regaining their mobility and social participation. This team of rehabilitation experts will help the patients to return to their former physical and social functioning, while medical and nursing care will be delegated to outpatient services and primary care physicians.

The cooperation between physical therapists and patients is intended to increase the patients’ motivation to perform the physical training. Therefore, the guidance of the physical therapists is based on a combination of the theoretical frameworks “self-determination theory” [78] and “theory of planned behavior” [79], and aims to improve the patients’ intention to perform the healthy behavior imparted by the GeRas program. In the GeRas program, patients will be supported in defining participation goals for their current life situation, the achievement of which is directly linked to physical improvements. In line with this, the physical therapists primarily discuss with the patients how physical exercise and walking can be integrated into their daily routine and support the implementation through weekly contacts. The multidisciplinary team identifies and addresses medical, social, and motivational barriers to physical exercise and social participation. A written dietary recommendation six weeks after discharge will be based on the patients’ dietary habits and changes in nutritional status. The unobtrusive nature of the written recommendations considers that one-on-one counseling by dietitians or nutritionists is rarely used and that dietary supplements are not covered by the health insurances in Germany. To overcome the limitations of the segregated health-care system, the involvement of a health insurance company facilitates the patients' social and medical care.

It is assumed that the need for assistance with care after discharge is often greater than anticipated by patients during discharge planning. The social workers of the discharging GR facilities and the health insurance company work together to optimize the (socio-) medical and nursing services during and after discharge, as well as to inform the patients and their relatives. The two case conferences after discharge from GR establish an organizational structure for the exchange of information between the professional groups involved and the different sectors of the health-care system. Community health services and primary care physicians are not yet involved for organizational reasons, but could be integrated in a next step after successful evaluation of the novel program.

Physical therapy as part of home-visits is very time-consuming. Compared to such home visits, eHealth interventions might be more time-efficient as less time is needed to travel to the patients’ home. Studies have shown that eHealth interventions have the potential to improve physical activity or balance in older adults during GR [80], but evidence on the feasibility and usability of eHealth interventions in geriatric patients living autonomously in their home environment is lacking. This hampers the implementation of eHealth solutions in geriatric settings [80]. The adoption of technology is lower among older adults compared to younger adults, although the use of technology, especially the use of computers, is also beneficial for people at a higher age [81, 82]. The WHO considers telemedicine applications as an opportunity to remotely monitor progress and to share relevant information between health-care providers, creating a continuous system of care with demonstrable improvements in health outcomes [83]. Furthermore, telemedicine can overcome barriers of distance and access to interventions, which is particularly beneficial for older adults living in rural areas [84]. In order to realize these advantages of telemedicine applications, the specific demands of older people and age-related issues in cognition, perception, and behavior need to be considered in the development of technological products and eHealth systems [85]. Positive experiences resulting from adapted, target-group-specific technologies can increase the likelihood that older adults will adopt innovative, less-familiar technologies [81]. In line with this, the intervention in the tablet IG of the GeRas study will be carried out by using an eHealth system that has been developed in cooperation with a peer group of geriatric patients.

The proposed GeRas program may represent an effective intervention to improve physical capacity and participation in geriatric patients after discharge to their home environment. The program can promote the success of the inpatient rehabilitation treatment and maintain the patients’ autonomy. By integrating the health insurance company, the program eliminates the sectoral segregation of inpatient and outpatient health care structures. The GeRas program has the potential to be implemented into existing health care plans.

### Trial status

The participant recruitment started in October 2022 and will run through April 30, 2024. At the time of submission (August 7, 2023), n = 122 participants have already been enrolled in the study.

### Supplementary Information

**Additional file 1.** 

## Data Availability

Anonymized datasets generated and analyzed as part of this study may be made available by the corresponding author upon reasonable request after completion of the study.
