# Supplementary figures and images for: Multimodal home-based rehabilitation intervention after discharge from inpatient geriatric rehabilitation (GeRas): study protocol for a multicenter randomized controlled trial
Source: BMC Geriatr. 2024 Jan 17;24:69. doi: 10.1186/s12877-023-04634-2 (PMC10795216; doi:10.1186/s12877-023-04634-2)

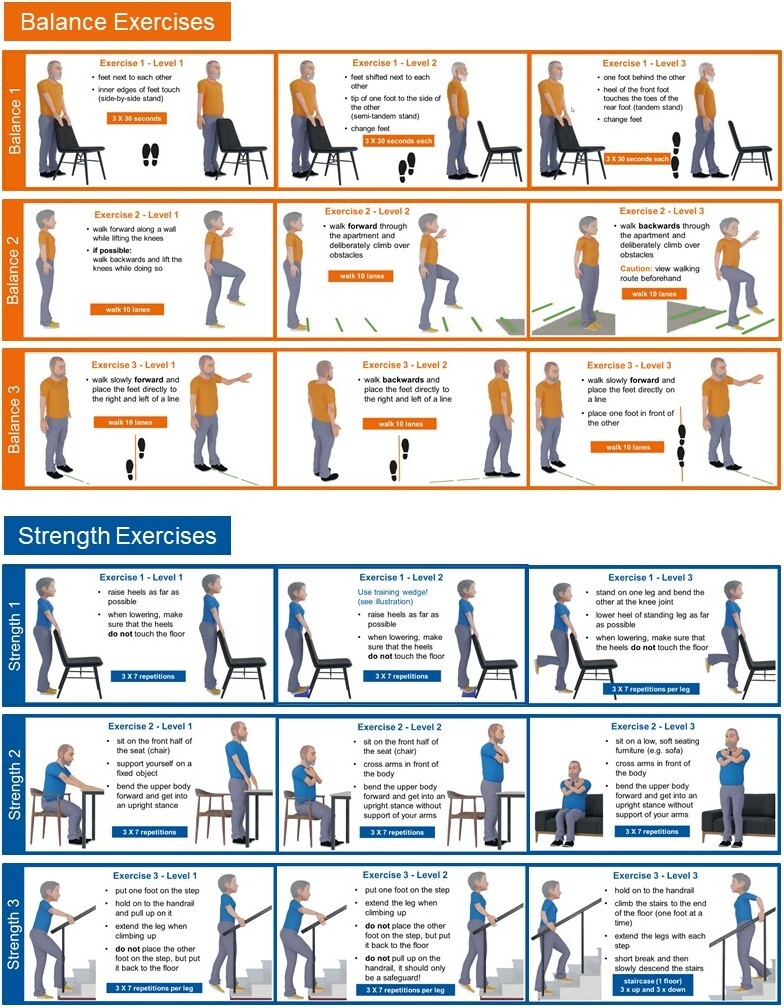

Supplement: Supplementary file 1 — Additional file 1. [file 12877_2023_4634_MOESM1_ESM.jpg]
